# Thumb posterior rotation deformity: a neglected deformity in children with thumb polydactyly

**DOI:** 10.3389/fped.2026.1894739

**Published:** 2026-08-12

**Authors:** ZiHua Lin, Lin Ye, LiuQing Liao, DiHao Tang, XueMei Lin, WeiZhe Shi, JianQun Wang, YiQiang Li

**Affiliations:** 1 Department of Orthopaedics Surgery, Heyuan Women & Children's Hospital and Health Institute, Heyuan, China; 2 Department of Pediatric Orthopaedics, Guangzhou Women and Children's Medical Center, Guangzhou Medical University, Guangzhou, China

**Keywords:** children, incidence, posterior rotation deformity, risk factor, thumb polydactyly

## Abstract

**Purpose:**

To investigate the incidence and risk factors of thumb posterior rotation deformity in children with thumb polydactyly.

**Methods:**

A retrospective analysis was conducted on the clinical data of 479 patients (521 thumbs, mean age 13.1 ± 12.7 months) with thumb polydactyly. Clinical data including gender, age, and affected side were collected. Clinical appearance photographs and x-ray images of all patients were obtained. Thumb posterior rotation deformity was determined on clinical appearance photographs. The ulnar deviation angle of the thumb was measured on anteroposterior thumb radiographs.

**Results:**

According to the Wassel classification, 13 thumbs (2.5%) were Type I, 55 thumbs (10.6%) Type II, 92 thumbs (17.7%) Type III, 186 thumbs (35.7%) Type IV, 138 thumbs (26.5%) Type V, 34 thumbs (6.5%) Type VI, and 3 thumbs (0.6%) Type VII. According to the Wu classification, 222 thumbs (42.6%) were type A, 27 thumbs (5.2%) were type B, 121 thumbs (23.2%) were type C, and 151 thumbs (29%) were type D. A total of 35 hands (6.7%) presented with varying degrees of thumb posterior rotation deformity, with a mean rotational angle of 14.1°±9.0° (range, 5°–30°). Wassel classification and Wu classification were correlated with the incidence of thumb posterior rotation deformity. Patients with thumb posterior rotation deformity had a significantly larger ulnar deviation angle of the thumb (19.3°±17.5°) than those without (11.4°±15.7°). Logistic regression analysis demonstrated that both the level and the type of the bifurcation of the duplicated thumb and ulnar deviation angle of the thumb were independent risk factors for the occurrence of thumb posterior rotation deformity (*P* < 0.05).

**Conclusions:**

Thumb posterior rotation deformity is a common malformation in pediatric thumb polydactyly. The incidence of thumb posterior rotation deformity is significantly increased in patients with Wassel types IV, V, and VI thumb polydactyly, and thumb ulnar deviation also significantly elevates the risk of thumb posterior rotation deformity.

## Introduction

Thumb polydactyly is the most common congenital hand malformation in children, with an incidence of approximately 0.08–1.4 per 1,000 live births (1). It is not merely an extra thumb, but is accompanied by a variety of complex deformities, such as thumb hypoplasia, joint instability, axial angular deformity, and nail malformation. Since thumb polydactyly significantly impairs hand function in children, early surgical intervention is generally required.

Detailed preoperative evaluation is of great significance for formulating the surgical treatment plan of thumb polydactyly. At present, preoperative evaluation for pediatric thumb polydactyly is mainly conducted through physical appearance assessment and x-ray examination. Multiple classification systems are also widely applied in clinical practice, including the Wassel classification (2), Chung classification (3), and Wu classification (4). Clinically, most physicians pay close attention to the angular deformity of the dominant ulnar thumb in thumb polydactyly and adopt various techniques for correction (5, 6).

In the author's clinical practice, another type of deformity has been identified: thumb posterior rotation deformity. This deformity is manifested as follows: when the dominant ulnar thumb is placed in the functional position at rest, its coronal plane fails to remain horizontal, presenting a posterior rotational position. Thumb posterior rotation deformity exerts a marked impact on the appearance of the dominant thumb. On the one hand, it aggravates the ulnar deviation of the dominant thumb in appearance; on the other hand, the thumb posterior rotation deformity becomes more obvious during active thumb dorsiflexion, making the thumb almost lie on the same plane as the other four fingers. This deformity may also have some impact on the function of the thumb.

This study retrospectively analyzes the clinical data of patients with thumb polydactyly treated in our institution, explores the incidence of thumb posterior rotation deformity among such cases, and attempts to analyze its etiological mechanism. The findings of this study will provide a new perspective for the preoperative evaluation of thumb polydactyly.

## Methods

A retrospective analysis was conducted on the clinical data of patients with thumb polydactyly admitted to Heyuan Women & Children's Hospital and Health Institute and Zengcheng Campus of Guangzhou Women and Children's Medical Center and from September 2024 to December 2025. This is an observational study. This study was approved by the Medical Ethics Committee of Heyuan Women & Children's Hospital and Health Institute (YXYJ-202620).

Inclusion criteria: 1) Diagnosed with thumb polydactyly; 2) Surgery performed between September 2024 and December 2025; 3) Complete clinical data available, including clinical photographs of the thumb and imaging data.

Exclusion criteria: 1) Previous surgical treatment performed at another hospital; 2) Incomplete clinical data; 3) Combined complex hand malformations, such as syndactyly, metacarpal or phalangeal dysplasia, ulnar polydactyly, and other associated anomalies.

According to the inclusion and exclusion criteria, a total of 479 cases (521 thumbs) were enrolled. Clinical data including gender, age, and affected side were collected. Clinical appearance photographs and x-ray images of all patients were obtained, and deformity evaluation was performed based on these imaging materials.

All cases of thumb polydactyly were classified according to the Wassel classification (2) and Wu classification (4). For patients with bilateral involvement, classification was performed separately for each side.

**Figure 1:**
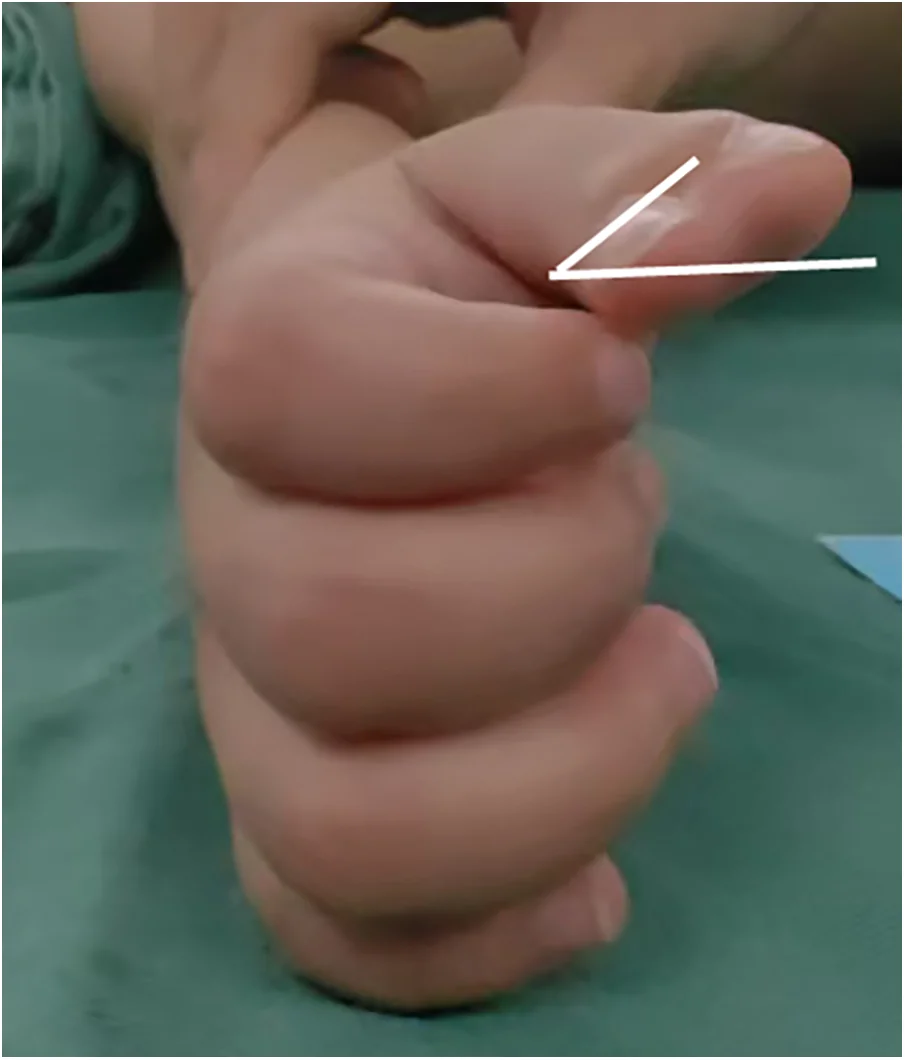
The measurement of the thumb posterior rotation deformity.

Preoperative clinical appearance photographs of the hand were taken to analyze the morphological features of polydactyly and determine the presence of thumb posterior rotation deformity. To minimize the influence of children's active movement on the evaluation of rotational deformity, all clinical appearance photographs were taken under general anesthesia. At rest state, the affected hand was placed in a functional position with the hand and wrist perpendicular to the surgical table. Anteroposterior and axial views were obtained. The presence of thumb posterior rotation deformity was judged on axial images. For patients with confirmed posterior rotation deformity, the rotational angle was further measured on axial images. The measurement method was as follows (Figure 1): On the axial view of the clinical appearance photograph, a first line was drawn perpendicular to the thumb axis along the dorsal aspect of the distal phalanx of the thumb, and then another straight line was drawn parallel to the surgical table. The angle formed by these two lines was defined as the thumb posterior rotation deformity angle. Two researchers measured the angles of all thumbs, and the mean value of them were used to perform statistical analysis. The normal reference value of this angle was less than 5°.

**Figure 2:**
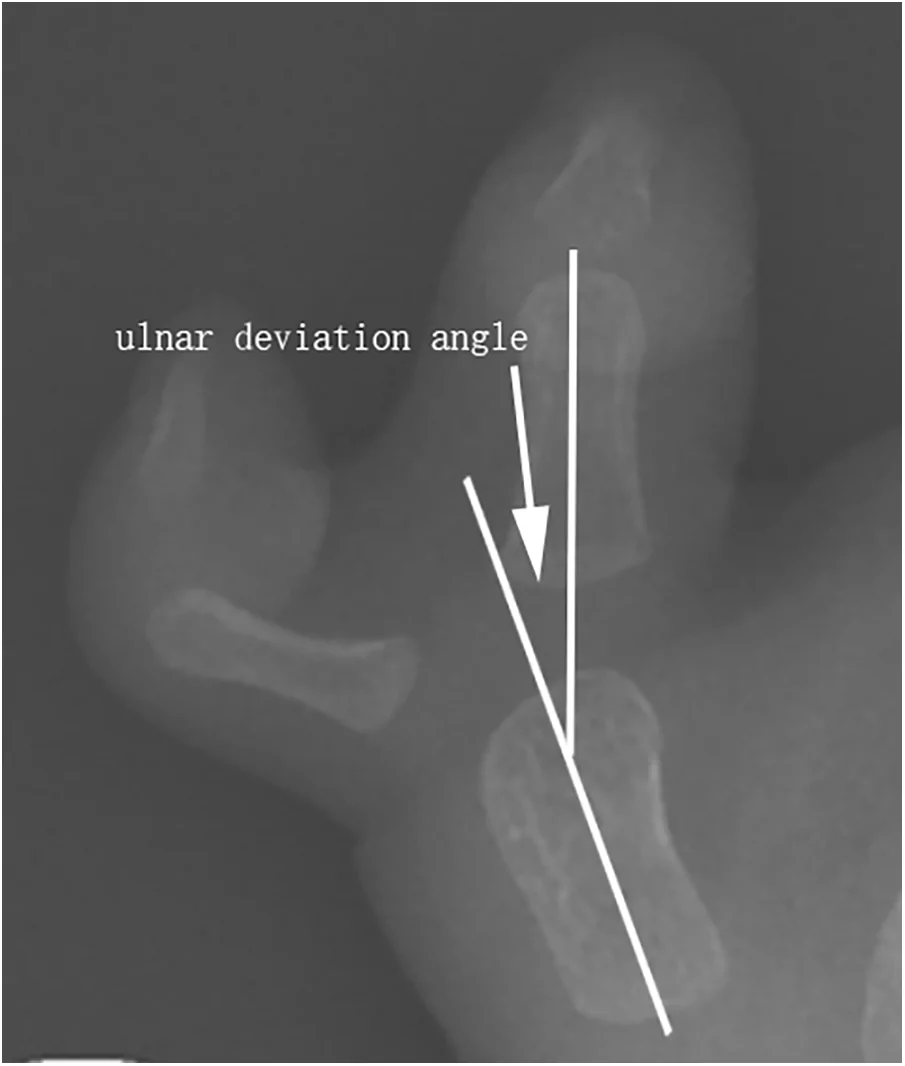
The measurement of the ulnar deviation angle of the thumb.

The ulnar deviation angle of the thumb was measured on anteroposterior thumb radiographs (Figure 2). Two longitudinal axes were drawn along the metacarpal bone and proximal phalanx respectively on the anteroposterior x-ray image, and the included angle between the two axes was defined as the thumb ulnar deviation angle. Two researchers measured the angles of all patients, and the mean value of them were used to perform statistical analysis. Normally, there was no obvious angular deformity of the metacarpophalangeal joint, with a baseline angle of 0°. An angle greater than 0° indicated ulnar deviation of the thumb, while an angle less than 0° indicated radial deviation.

SPSS 22.0 software was used for statistical analysis. A *P*-value less than 0.05 was considered statistically significant. Numerical variables such as age, rotational angle and metacarpophalangeal joint angle were analyzed using the t-test or one-way analysis of variance. Categorical variable including gender, affected side and Wassel classification were compared between groups using the Chi-square test. Logistic analysis was used to determine the risk factors of thumb posterior rotation deformity. Because there were zero events, and interaction effect between Wassel and Wu classification when we perform logistic regression. To address this problem, we set two new categories based on Wassel and Wu classifications. The first was the level of the bifurcation of the duplicated thumb (distal phalanx, interphalangeal joint, proximal phalanx, metacarpophalangeal joints, metacarpal bone, carpometacarpal joints); the second was the type of the connection of the duplicated thumb with the ulnar main thumb (Joint, bone or unossified cartilage, soft tissue).

## Results

Among the 479 enrolled patients (521 thumbs), the mean age was 13.1 ± 12.7 months (range, 2.0–106.5 months). There were 179 females (37.4%) and 300 males (62.6%). In terms of affected side, 170 cases (35.5%) were left-sided, 267 cases (55.7%) were right-sided, and 42 cases (8.8%) were bilateral.

According to the Wassel classification: 13 thumbs (2.5%) were Type I, 55 thumbs (10.6%) Type II, 92 thumbs (17.7%) Type III, 186 thumbs (35.7%) Type IV, 138 thumbs (26.5%) Type V, 34 thumbs (6.5%) Type VI, and 3 thumbs (0.6%) Type VII.

According to the Wu classification: 33 thumbs (6.3%) were Type A1, 169 (32.4%) Type A2, 18 (3.5%) Type A3, 2 (0.4%) Type A4, 6 (1.2%) Type B1, 16 (3.1%) Type B2, 5 (1.0%) Type B3, 1 (0.2%) Type C1, 26 (5.0%) Type C2, 94 (18.0%) Type C3, 23 (4.4%) Type D1, 68 (13.1%) Type D2, 58 (11.1%) Type D3, and 2 (0.4%) Type D4. Overall, 222 thumbs (42.6%) were type A, 27 thumbs (5.2%) were type B, 121 thumbs (23.2%) were type C, and 151 thumbs (29%) were type D.

Based on the diagnostic criteria of thumb posterior rotation deformity, a total of 35 hands (6.7%) presented with varying degrees of thumb posterior rotation deformity, with a mean rotational angle of 14.1°±9.0° (range, 5°–30°). The intraclass correlation coefficient (ICC) was 0.964. Age, gender and affected side showed no significant correlation with the occurrence of thumb posterior rotation deformity (*P* > 0.05) (Table 1).

**Table 1:** The clinical data of the patients with and without thumb posterior rotation deformity.

Posterior rotation deformity	N	Group	Without	With	t/Z/*χ*²	P
N	521		486 (93.3%)	35 (6.7%)		
Age (months)	479		13.0 ± 12.6	11.2 ± 7.6	0.383	0.702^b^
Gender	479	female	170 (95%)	9 (5.0%)	1.544	0.214
		male	276 (92%)	24 (8%)		
Side	521	left	160 (94.1%)	10 (5.9%)	0.281	0.869
		Right	248 (92.9%)	19 (7.1%)		
		Bilateral	78 (92.9%)	6 (7.1%)		
Wassel classification	521	I	13 (100%)	0 (0)	13.8	0.032^a^
		II	53 (96.4%)	2 (3.6%)		
		III	91 (98.9%)	1 (1.1%)		
		IV	168 (90.3%)	18 (9.7%)		
		V	129 (93.5%)	9 (6.5%)		
		VI	30 (88.2%	4 (11.8%)		
		VII	2 (66.7%)	1 (33.3%)		
Wu classification	521	A	207 (93.2%)	15 (6.8%)	50.944	<0.001
		B	17 (63%)	10 (37%		
		C	111 (91.7%)	10 (8.3%)		
		D	151 (100%)	0 (0)		
Level of the bifurcation		DP&IJ	66 (97.1%)	2 (2.9%)	-	0.015^a^
		PP	91 (98.9%)	1 (1.1%)		
		MP	169 (89.9%)	19 (10.1)		
		MB	130 (93.5%)	9 (6.5%)		
		CJ	30 (88.2%)	4 (11.8%)		
Type of bifurcation		JB	207 (93.2%)	15 (6.8%)	21.782	<0.001
		BB	128 (86.5%)	20 (13.5%)		
		SB	151 (100%)	0 (0)		
Ulnar deviation angle (°)	521		11.4 ± 15.7	19.3 ± 17.5	3.195	0.001^b^

*N* = 479: analysis based on patient-level; *N* = 521: analysis based on thumb-level.

Level of bifurcation: distal phalanx (DP), interphalangeal joint (IJ), proximal phalanx (PP), metacarpophalangeal joints (MP), metacarpal bone (MB), carpometacarpal joints (CJ).

Type of bifurcation: Joint bifurcation (JB), bone or unossified cartilage bifurcation(BB), soft tissue bifurcation (SB).

a Fisher's exact test.

b non-parametric test.

Wassel classification was correlated with the incidence of thumb posterior rotation deformity. The incidence of thumb posterior rotation deformity in Wassel Type IV, V and VI was significantly higher than that in Type I, II and III (*P* < 0.05) (Table 1).

Wu classification was also correlated with the incidence of thumb posterior rotation deformity. The incidence of thumb posterior rotation deformity in Type A, B and C was significantly higher than that in Type D (*P* < 0.001) (Table 1).

The mean ulnar deviation angle of the thumb was 12.0°±15.9° (range, −78° to 60°). The intraclass correlation coefficient (ICC) was 0.917. Ulnar deviation angle of the thumb was significantly associated with thumb posterior rotation deformity (*P* = 0.005). Patients with thumb posterior rotation deformity had a significantly larger ulnar deviation angle of the thumb (19.3°±17.5°) than those without (11.4°±15.7°) (Table 1). It indicated that a larger ulnar deviation angle of the thumb was associated with a higher probability of developing thumb posterior rotation deformity. Pearson correlation analysis showed that among the 35 hands with posterior rotation deformity, there was no significant correlation between ulnar deviation angle of the thumb and rotational angle (r = 0.17, *P* = 0.33).

Logistic regression analysis demonstrated that both the level and the type of the bifurcation of the duplicated thumb and ulnar deviation angle of the thumb were independent risk factors for the occurrence of thumb posterior rotation deformity (*P* < 0.05) (Table 2). Among patients with thumb polydactyly, the incidence of thumb posterior rotation deformity is higher when the accessory thumb is located at the proximal thumb or the duplicated thumb is connected via joint, bone or cartilage. Additionally, the greater the ulnar deviation angle of the thumb, the higher the incidence of thumb supination deformity.

**Table 2:** Risk factors for thumb posterior rotation deformity.

Factors	CE	SE	Wald	P	EXP(CE)	95% CI for EXP(CE)
Wassel classification	0.464	0.184	6.346	0.012	1.590	1.108, 2.281
Wu classification	−0.351	0.162	4.695	0.030	0.704	0.513, 0.967
Ulnar deviation angle	0.026	0.012	4.809	0.028	1.026	1.003, 1.051

CE, co-efficient; SE, standard error; CI, confidence interval.

## Discussion

This study demonstrated that thumb posterior rotation deformity is a common malformation in thumb polydactyly, with an incidence rate of 6.7%. Currently, no relevant studies have reported the incidence of this deformity in thumb polydactyly. Thus, we propose a hypothesis that the abnormal embryonic development of the hand might lead to this deformity. Previous studies have shown that the development of the hand is an extremely complex process: limb buds emerge at the 4th week of embryonic development, and the forearm and hand plate can be distinguished on the 33rd day. The five fingers gradually separate starting from the 38th day of embryonic development and are basically formed by the 56th day (7, 8). Therefore, thumb polydactyly may occur between the 38th and 56th days of embryonic development. Any gene mutation or disturbance of microenvironmental factors may lead to abnormal finger separation, resulting in the formation of redundant digits. The development of the thumb is more complex: in the early embryonic stage, the five fingers separate from the hand plate and lie on the same plane. During subsequent embryonic development, the upper limb rotates along its longitudinal axis (8), and the thumb gradually develops to the normal neutral position. In patients with thumb posterior rotation deformity, dysplasia of upper limb rotation during embryonic development may occur, leading to the failure of the thumb to rotate to its normal position.

**Figure 3:**
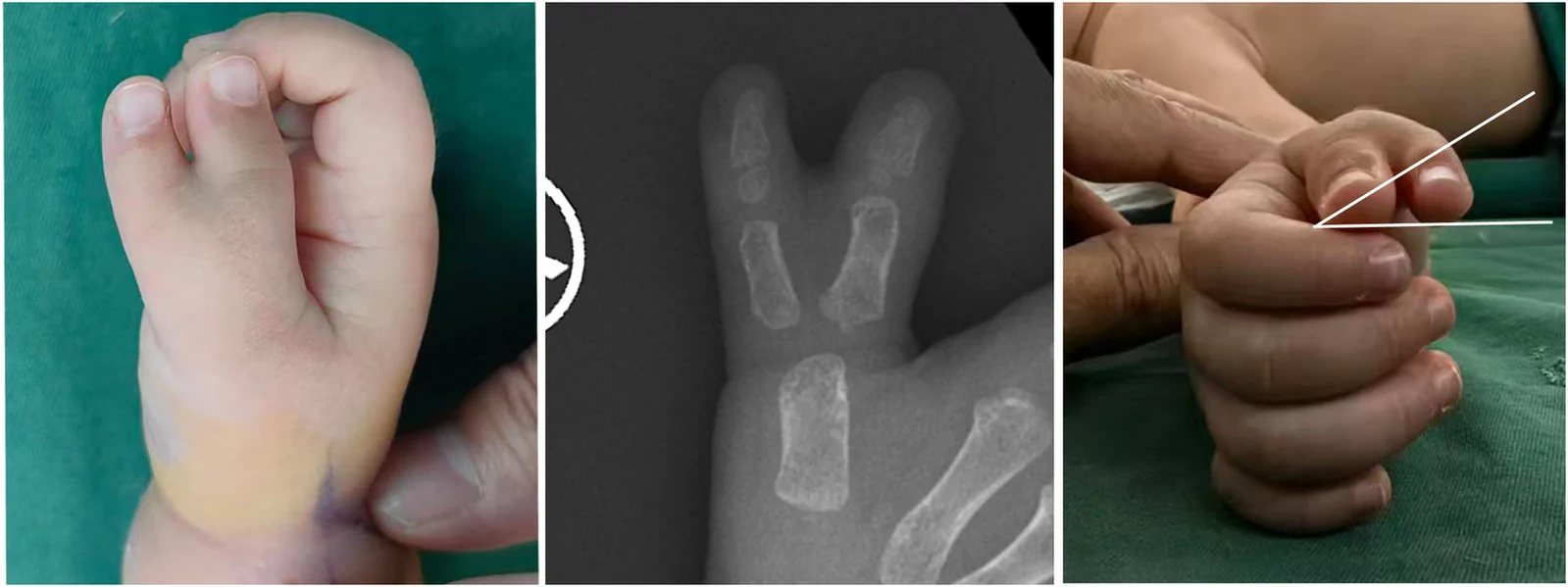
A patient (16 months, female) with Wassel type IV thumb polydactyly on the right side. She had significant thumb posterior rotation deformity, and the rotational angle is 30°.

**Figure 4:**
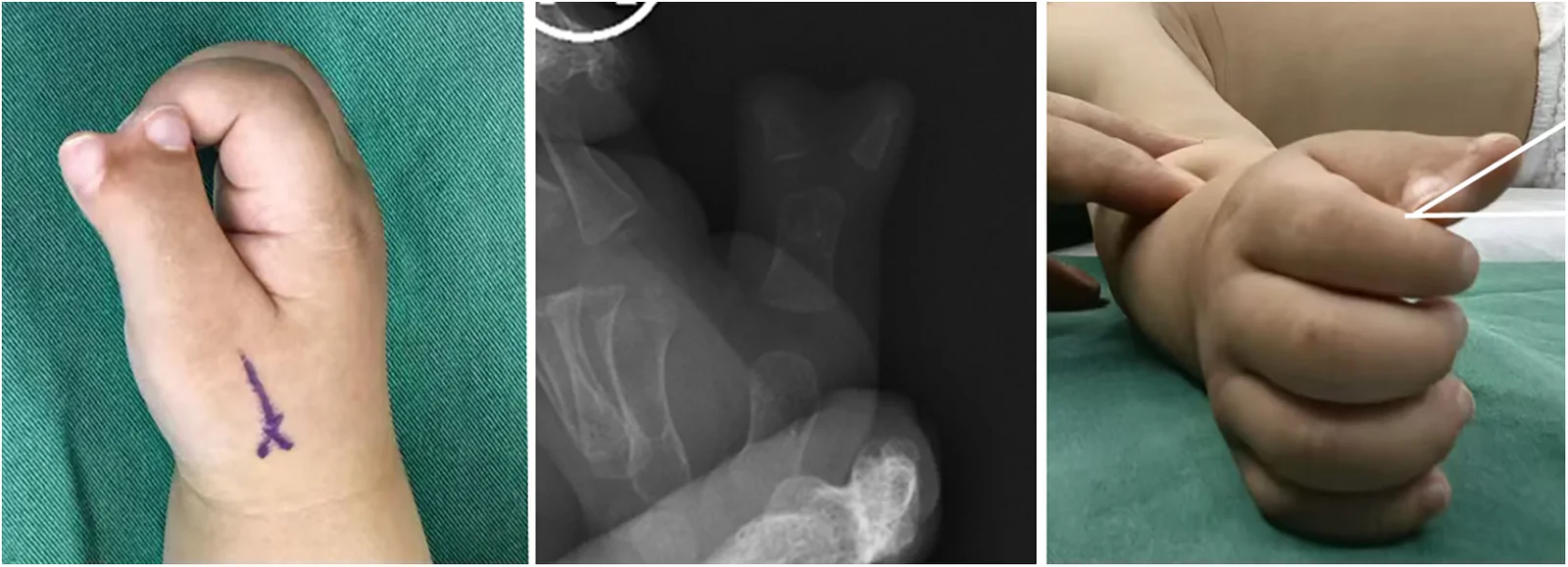
A patient (11 months, male) with Wassel type III thumb polydactyly on the right side. He had significant thumb posterior rotation deformity, and the rotational angle is 30°.

This study found that the incidence of thumb posterior rotation deformity is higher when the accessory thumb is located at the proximal thumb or the duplicated thumb is connected via joint, bone or cartilage. It means that thumb polydactyly of Wassel types IV, V, and VI carried a higher risk of thumb posterior rotation deformity (Figure 3), suggesting that thumb rotational deformity mainly occurs at the proximal part of the thumb. Previous studies have reported that thumb rotation mainly occurs at the carpometacarpal joint (9). In a normal thumb, the thumb phalanx is horizontal in the neutral position, with the distal thumb touching the distal index finger; maintenance of this position mainly relies on the functions of the abductor pollicis brevis, flexor pollicis brevis, and opponens pollicis. Normally, the insertions of these muscles are located on the radial and palmar sides around the metacarpophalangeal joint of the thumb (10). In Wassel types IV, V, and VI thumb polydactyly, the redundant thumb bifurcates from the metacarpal bone or metacarpophalangeal joint. The presence of the radial redundant digit alters the anatomical position of the abductor pollicis brevis, flexor pollicis brevis, and opponens pollicis (11, 12), with their insertions attached to the radial redundant thumb, which may cause insufficient opposition of the dominant thumb (10). Although thumb posterior rotation deformity occurs more frequently in patients with Wassel type IV, V and VI duplicated thumbs, a small number of Wassel type III duplicated thumbs may occasionally present with this deformity (Figure 4).

This study indicated that the greater the ulnar deviation angle of the thumb, the higher the incidence of thumb posterior rotation deformity. Ulnar deviation angle of the thumb is a common malformation in thumb polydactyly (13, 14), characterized by an ulnar-angled deviation between the axes of the proximal and distal phalanges of the ulnar dominant thumb and the metacarpal bone. This deformity is a key factor affecting the appearance and stability of the dominant thumb, and a larger angulation leads to greater instability of the dominant thumb. In such polydactyly cases, the dominant thumb forms a certain angle with the radial duplicated thumb. As the ulnar deviation angle increases, the distal insertions of the abductor pollicis brevis, flexor pollicis brevis, and opponens pollicis move further away from the metacarpophalangeal joint of the dominant thumb, which may consequently increase the occurrence of thumb posterior rotation deformity. Interestingly, correlation analysis in this study showed no significant correlation between the degree of thumb ulnar deviation and the severity of dominant thumb posterior rotation deformity.

The management of thumb posterior rotation deformity mainly involves simultaneous correction during resection of the duplicated thumb. Since thumb posterior rotation deformity mainly occurs proximally, the treatment can focus on the metacarpophalangeal joint and metacarpal levels. When resecting the radial duplicated thumb, the surgeon must carefully preserve the insertions of the abductor pollicis brevis, flexor pollicis brevis, and opponens pollicis at the metacarpophalangeal joint of the duplicated digit, and finally reattach them to the metacarpophalangeal joint of the ulnar dominant thumb. For patients with ulnar deviation deformity, wedge osteotomy of the metacarpal bone should be performed, combined with derotation and fixation of the distal osteotomy segment to correct thumb posterior rotation deformity. Currently, there is no unified consensus on the indications for metacarpal osteotomy worldwide (15, 16). In our opinion, metacarpal wedge osteotomy is indicated when the ulnar dominant thumb has an ulnar deviation deformity of more than 10° after resection of the radial duplicated thumb.

In conclusion, thumb posterior rotation deformity is a common malformation in pediatric thumb polydactyly. Wassel classification, Wu classification and ulnar deviation angle of the thumb are risk factors for thumb posterior rotation deformity. The incidence of thumb posterior rotation deformity is significantly increased in patients with Wassel types IV, V, and VI thumb polydactyly, and thumb ulnar deviation also significantly elevates the risk of thumb posterior rotation deformity.

## Data Availability

The raw data supporting the conclusions of this article will be made available by the authors, without undue reservation.
